# Chromosome-scale comparative sequence analysis unravels molecular mechanisms of genome dynamics between two wheat cultivars

**DOI:** 10.1186/s13059-018-1477-2

**Published:** 2018-08-17

**Authors:** Anupriya Kaur Thind, Thomas Wicker, Thomas Müller, Patrick M. Ackermann, Burkhard Steuernagel, Brande B. H. Wulff, Manuel Spannagl, Sven O. Twardziok, Marius Felder, Thomas Lux, Klaus F. X. Mayer, International Wheat Genome Sequencing Consortium, Beat Keller, Simon G. Krattinger

**Affiliations:** 1 Department of Plant and Microbial Biology, University of Zurich, Zollikerstrasse 107, Zurich, Switzerland; 2 John Innes Centre, Norwich Research Park, Norwich, UK; 3 Helmholtz Zentrum Munich, Munich, Germany; 4 School of Life Sciences, Technical University Munich, Munich, Germany; 5 College of Science, King Saud University, Riad, Kingdom of Saudi Arabia; 6 International Wheat Genome Sequencing Consortium (IWGSC), 2841 NE Marywood Ct, Lee’s Summit, MO 64086 USA; 7 King Abdullah University of Science and Technology, Thuwal, Kingdom of Saudi Arabia

**Keywords:** Genome diversity, Structural variation, High-quality assembly, Wheat

## Abstract

**Background:**

Recent improvements in DNA sequencing and genome scaffolding have paved the way to generate high-quality de novo assemblies of pseudomolecules representing complete chromosomes of wheat and its wild relatives. These assemblies form the basis to compare the dynamics of wheat genomes on a megabase scale.

**Results:**

Here, we provide a comparative sequence analysis of the 700-megabase chromosome 2D between two bread wheat genotypes—the old landrace Chinese Spring and the elite Swiss spring wheat line ‘CH Campala *Lr22a*’. Both chromosomes were assembled into megabase-sized scaffolds. There is a high degree of sequence conservation between the two chromosomes. Analysis of large structural variations reveals four large indels of more than 100 kb. Based on the molecular signatures at the breakpoints, unequal crossing over and double-strand break repair were identified as the molecular mechanisms that caused these indels. Three of the large indels affect copy number of NLRs, a gene family involved in plant immunity. Analysis of SNP density reveals four haploblocks of 4, 8, 9 and 48 Mb with a 35-fold increased SNP density compared to the rest of the chromosome. Gene content across the two chromosomes was highly conserved. Ninety-nine percent of the genic sequences were present in both genotypes and the fraction of unique genes ranged from 0.4 to 0.7%.

**Conclusions:**

This comparative analysis of two high-quality chromosome assemblies enabled a comprehensive assessment of large structural variations and gene content. The insight obtained from this analysis will form the basis of future wheat pan-genome studies.

**Electronic supplementary material:**

The online version of this article (10.1186/s13059-018-1477-2) contains supplementary material, which is available to authorized users.

## Background

Bread wheat (*Triticum aestivum*) was the most widely grown cereal crop in 2016. It serves as a staple food for over 30% of the world’s population and provides ~ 20% of the globally consumed calories [1]. Wheat is a young allopolyploid species with a genome size of 15.4–15.8 Gb, of which more than 85% is made up of highly repetitive sequences [2]. The allopolyploid genome arose through two recent, natural polyploidization events that involved three diploid grass species. The first hybridization event occurred 0.58 to 0.82 million years ago [3] between the A genome donor wild einkorn (*Triticum urartu*) and a yet unidentified B genome donor that was a close relative of *Aegilops speltoides*. This hybridization created wild tetraploid emmer wheat (*Triticum turgidum* ssp. *dicoccoides*; AABB genome) [4]. A second natural hybridization between domesticated emmer and wild goatgrass (*Aegilops tauschii*; DD genome) resulted in the formation of hexaploid bread wheat (AABBDD genome) around 10,000 years ago [5]. The domestication of tetraploid emmer and the limited number of hybridization events with *Ae. tauschii* represent bottlenecks that resulted in a significant reduction of genetic diversity within the bread wheat gene pool. Natural gene flow between bread wheat and its wild and domesticated relatives as well as artificial hybridizations with diverse grass species partially compensated for this loss in diversity [3, 6].

The size, repeat content and polyploidy of the bread wheat genome have represented major challenges for the generation of a high-quality reference assembly. The first ‘early’ whole genome assemblies of hexaploid wheat and its diploid wild relatives were based on short-read sequencing approaches. These assemblies provided an insight into the gene space of wheat, but they were highly fragmented and incomplete [7–10]. The first notable high-quality sequence assembly of wheat was produced from the 1-gigabase chromosome 3B of the hexaploid wheat landrace Chinese Spring. For this, 8452 ordered bacterial artificial chromosomes (BACs) were sequenced and assembled, which resulted in a highly contiguous assembly (N50 = 892 kb) [11, 12]. More recent whole-genome shotgun assemblies had improved contiguousness compared to the ‘early’ assemblies (N50 = 25–232 kb) [13–15], but they still did not allow comparison of the structures of wheat chromosomes on a megabase-scale.

Several recent technological and computational improvements, however, provide a basis to generate de novo assemblies of complex plant genomes with massively improved scaffold lengths and completeness. These advancements include (*i*) the integration of whole-genome shotgun libraries of various insert sizes [16] or the use of long-read sequencing technologies such as single-molecule real-time sequencing (SMRT) [17] or nanopore sequencing [18], (*ii*) the improvement of scaffolding by using chromosome conformation capture technologies [19–23] or optical maps [24] and (*iii*) the improvement of assembly algorithms [4]. With the use of some of these novel approaches, a near complete reference assembly of Chinese Spring (IWGSC RefSeq v1.0) with a scaffold N50 of 22.8 Mb was recently generated [25]. Chinese Spring is an old landrace that was selected for sequencing because it was used in a number of cytogenetic studies, which has resulted in the generation of many important genetic resources from this wheat line, including chromosome deletion lines [26] and aneuploid lines [27].

The completion of the IWGSC RefSeq v1.0 assembly lays the foundation to study the genetic diversity within and between different wheat species and cultivars. The understanding of this genetic variation will provide an insight into wheat genome dynamics and its impact on agronomically important traits. The continuum of genetic variation ranges from single nucleotide polymorphisms (SNPs) to megabase-sized rearrangements that can affect the structure of entire chromosomes [28]. Due to the absence of high-quality wheat genome assemblies, previous comparative analyses were limited in the size of structural rearrangements that could be assessed and, typically, structural variants of a few base pairs up to several kilobases were analyzed [29, 30]. Consequently, a comprehensive assessment of the extent of large structural rearrangements and their underlying molecular mechanisms is still lacking.

Here, we report on a chromosome-wide comparative analysis of the ~ 700-Mb chromosome 2D between the two hexaploid wheat lines Chinese Spring and ‘CH Campala *Lr22a*’. CH Campala *Lr22a* is a backcross line that was generated to introgress *Lr22a*, a gene that provides resistance against the fungal leaf rust disease, into the genetic background of the elite Swiss spring wheat cultivar CH Campala [31]. We previously generated a high-quality de novo assembly from isolated chromosome 2D of CH Campala *Lr22a* by using short-read sequencing in combination with Chicago long-range scaffolding [32]. The resulting assembly had a scaffold N50 of 9.76 Mb. Here, we compared this high-quality assembly to chromosome 2D of the Chinese Spring IWGSC RefSeq v1.0 assembly. In particular, the focus of our study was on the identification and quantification of large structural variations (SVs). The comparative analysis of the 2D chromosome showed a high degree of collinearity along most of the chromosome, but also revealed SVs such as InDels and copy number variation (CNV). In addition, we found haploblocks with greatly increased SNP densities. We analyzed these SVs and gene presence/absence polymorphisms in detail and manually validated them to distinguish true SVs from artefacts that were due to mis-assembly or annotation problems.

## Results

### Two-way comparison of Chinese spring and CH Campala *Lr22a* allows identification of large structural variations

Previously, 10,344 sequence scaffolds were produced from isolated chromosome 2D of CH Campala *Lr22a* by using Chicago long-range linkage [21, 32]. To construct a CH Campala *Lr22a* pseudomolecule, we anchored these scaffolds to the IWGSC RefSeq v1.0 chromosome 2D using BLASTN (see “Methods”). In the resulting CH Campala *Lr22a* pseudomolecule, 7617 scaffolds were anchored, of which 7314 were smaller than 5 kb and 90 were larger than 1 Mb in size. The pseudomolecule had a scaffold N50 of 8.78 Mb (N90 of 1.89 Mb) and represented 98.92% of the total length of the initial assembly. The CH Campala *Lr22a* pseudomolecule has a total length of 563 Mb whereas the IWGSC RefSeq v1.0 2D pseudomolecule is 651 Mb in length. It was previously found that repetitive sequences were collapsed and less complete in the Chicago assembly, which explains the smaller size of the CH Campala *Lr22a* pseudomolecule compared to the IWGSC RefSeq v1.0 pseudomolecule [32]. In total, 6018 high confidence (HC) genes were annotated in Chinese Spring [25] and 5883 HC genes in CH Campala *Lr22a* (see “Methods”). Of the 5883 CH Campala *Lr22a* HC genes, 45 genes were located on short scaffolds that contained no other gene. Gene annotation and collinearity will be discussed in detail in a following section.

To identify large InDels, we compared the Chinese Spring and CH Campala *Lr22a* pseudomolecules in windows of 10 Mb and constructed dot plots. Here, we focused only on InDels larger than 100 kb because such SVs could not be identified with previous whole-genome assemblies. In total, we found 26 putative InDels, which were manually validated by evaluating the upstream and downstream sequences for the presence of ‘Ns’ at the breakpoints. If Ns were found exactly at the breakpoints on both sides of an InDel, we considered it a false positive that was most likely due to the incorrect placement of a scaffold in either of the pseudomolecules. Based on this criterion, we discarded 22 of the 26 candidate InDels. Three of the remaining four InDels showed good sequence quality and had clear breakpoints at both ends with no Ns. These true InDels were 285, 494 and 765 kb in size. An additional 677-kb InDel had a clear break only at one end and Ns on the other end. Interestingly, three of the four large InDels showed CNV for nucleotide binding site-leucine-rich repeat (NLR) genes.

Various molecular mechanisms have been described that lead to SVs. For example, unequal crossing over can occur in regions with extensive sequence similarity. On the other hand, non-homologous end-joining (NHEJ) is associated with DNA repair in regions with no or low sequence similarity. Other causes of SVs include double-strand break (DSB) repair via single-strand annealing or synthesis-dependent strand annealing mechanisms, transposable element (TE)-mediated mechanisms and replication-error mechanisms [33–36]. These mechanisms have been well studied in humans, but in plants our understanding of the molecular causes of SVs is limited [33]. To decipher the mechanistic bases of the observed SVs, the sequence of the SV as well as of their flanking regions were analyzed to identify signature sequence motifs that could point to the underlying molecular mechanism (e.g. DNA repair-, recombination- or replication-associated mechanisms).

### Unequal crossing over is the likely cause of a 285-kb deletion in Chinese spring

**Fig. 1:**
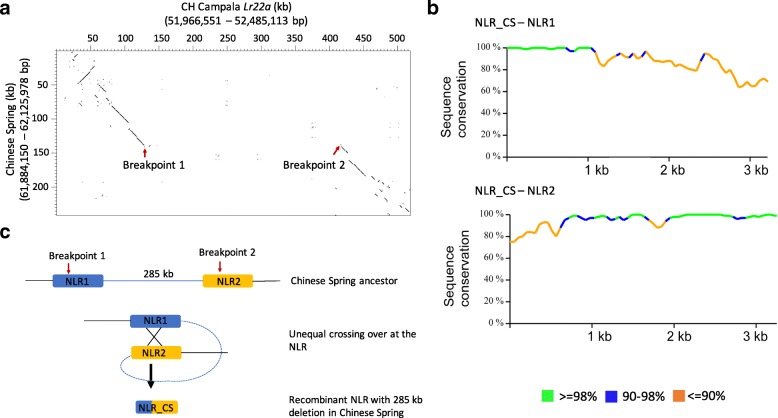
Unequal crossing over resulted in a 285-kb deletion in Chinese Spring. **a** Dot plot of a 525-kb segment from CH Campala *Lr22a* against the corresponding 280-kb segment from Chinese Spring. The breakpoints of the 285-kb deletion are indicated by *red arrows*. The numbers in brackets refer to the positions of the selected region on the respective pseudomolecule. **b** Pairwise alignment of the Chinese Spring NLR with the two CH Campala *Lr22a* NLRs shows putative recombination breakpoints that led to the formation of the Chinese Spring NLR. **c** Proposed model for molecular events that led to a 285-kb deletion in Chinese Spring. An unequal crossing over event involving two NLR genes (shown in *blue* and *orange*) led to the formation of the recombinant NLR in Chinese Spring which shares sequence homology with NLR1 (*blue*) and NLR2 (*yellow*) and a deletion of the intervening 285 kb sequence

Sequence comparison revealed an InDel of 285 kb on the short chromosome arm (Fig. 1a). We extracted and checked the sequences 5 kb upstream and downstream of the breakpoints for the presence of TEs or genes (or any kind of repeated sequence) that could have served as a template for unequal crossing over. Unequal crossing over occurs frequently at repeated sequences that are in the same orientation, leading to duplications or deletions of the region between the two repeats [37]. Indeed, the breakpoints of the InDel contained two NLR genes that shared 96–98% nucleotide identity in CH Campala *Lr22a*. In contrast, Chinese Spring only carried a single NLR copy (Fig. 1). Thus, it is possible that an unequal crossing over between the two genes occurred in an ancestor of Chinese Spring, leading to the loss of the 285-kb segment between the two NLRs.

In order to test this hypothesis, we further analyzed the NLRs that were present at the breakpoint of CH Campala *Lr22a* and Chinese Spring. Interestingly, the 5′ region of the Chinese Spring gene showed greater sequence similarity to NLR1 of CH Campala *Lr22a*, whereas the 3′ region was more similar to NLR2 (Fig. 1b). This suggests that these NLRs (NLR1 and NLR2) were indeed the template for an unequal crossing over in an ancestor of Chinese Spring (Fig. 1c). The corresponding 285-kb segment in CH Campala *Lr22a* only contained repetitive sequences and did not carry any genes.

### Double-strand break repair likely mediated a large 494-kb deletion

**Fig. 2:**
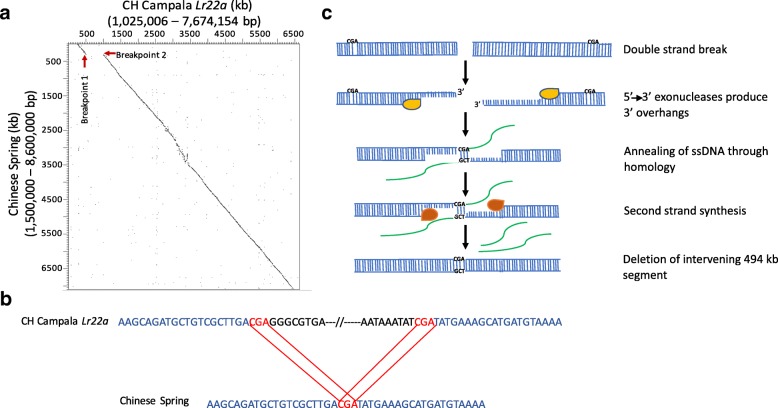
Double-strand break repair is responsible for the deletion of a 494-kb segment in Chinese Spring. **a** Dot plot of a 6.6-Mb scaffold of CH Campala *Lr22a* against the corresponding segment from Chinese Spring. The breakpoints are indicated by *red arrows*. The numbers in brackets refer to the positions of the selected region on the respective pseudomolecule. **b** Presence of DSB signatures (CGA triplet, *red*) with two copies in CH Campala *Lr22a* and one in Chinese Spring. The conserved sequence is shown in *blue* and the 494-kb sequence that is deleted in Chinese Spring but present in CH Campala *Lr22a* is indicated in *black*. **c** The proposed model for the deletion of the 494-kb segment in Chinese Spring through DSB repair by the single-strand annealing pathway, where the *yellow enzyme* is the exonuclease, *green strands* are the overhangs and the *orange colour* represents the replication complex

The second SV was located on a CH Campala *Lr22a* scaffold of 6.6 Mb in size (Fig. 2a). We could precisely identify the breakpoints based on the sequence alignment of the two wheat lines. Unlike the case described above, the upstream and downstream sequences contained no obvious sequence template or a typical TE insertion or excision pattern [34] that could have led to a large deletion by unequal crossing over. However, the breakpoints of the InDel contained typical signatures of DSB repair. In CH Campala *Lr22a* the nucleotide triplet CGA was repeated at both ends of the breakpoint whereas Chinese Spring had only one copy of the CGA triplet (Fig. 2b). The proposed model for this 494-kb deletion is that it was caused through a DSB that was repaired by the single-strand annealing pathway (Fig. 2c). After the DSB that could have occurred anywhere on the 494-kb segment in Chinese Spring, 3′ overhangs were produced by exonucleases. Various studies in yeast have shown that these overhangs can be many kilobases in size [38–40] and, due to high conservation of DSB repair pathways [41], it is expected that plants would have a similar DSB repair mechanism. In the case described here, we propose that exonucleases produced overhangs of 200–250 kb, which were then repaired by non-conservative homologous recombination repair (HRR). For this, the generated 3′ overhangs annealed in a place of complementary micro-homology, which are typically a few base pairs in size (CGA triplet in this case) [42]. After annealing of the matching motifs, second strand synthesis took place and the overhangs were removed, leading to the observed deletion of the 494-kb sequence in Chinese Spring (Fig. 2c). This 494-kb segment in CH Campala *Lr22a* contained eight genes coding for an NLR, a serine/threonine protein kinase, a zinc finger-containing protein, a transferase, two cytochrome P450s and two proteins of unknown function. BLAST analysis of these eight genes against the IWGSC RefSeq v1.0 pseudomolecules revealed that the homoeologous segments on the A and B genomes were retained. In other words, the deletion of these eight genes might not have led to a deleterious effect because the homoeologous gene copies on the other two sub-genomes compensate for the D genome deletion. It has been reported that polyploid species show a higher plasticity compared to diploid species and that they are able to buffer large insertions and deletions on one particular sub-genome [43].

### Large diverse haploblocks indicate recurrent gene flow from distant relatives

**Fig. 3:**
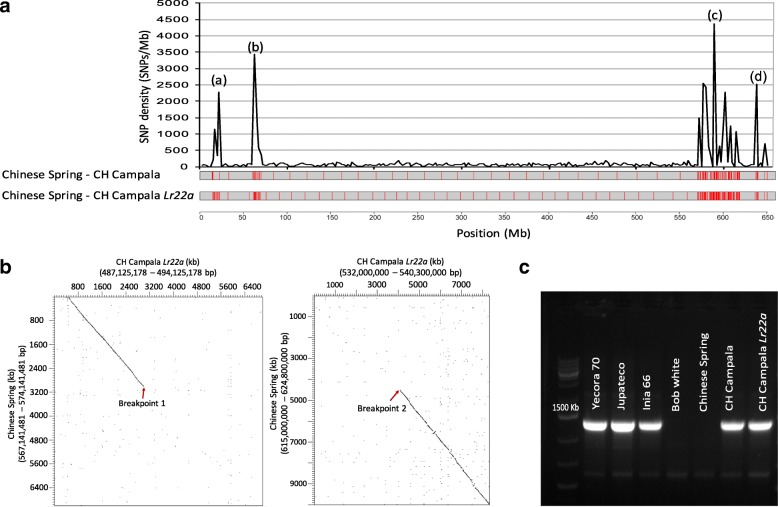
Identification of four diverse haploblocks with increased SNP density. **a** Single nucleotide polymorphism (SNP) density between Chinese Spring and CH Campala *Lr22a* in a sliding window of 2.5 Mb. The numbers refer to the position in megabases along the chromosome 2D of the Chinese Spring. The four diverse haploblocks are indicated with letters *a*, *b*, *c* and *d*. **b** Dot plot of Chinese Spring and CH Campala *Lr22a* showing the left and right breakpoints of the large haploblock *c*. The sequence adjacent to the haploblock shows a high degree of sequence conservation in intergenic regions whereas the sequence similarity was very low in the haploblock region. The numbers in brackets refer to the positions of the selected region on the respective pseudomolecule. **c** PCR amplification using an introgression-specific primer pair designed on the left breakpoint of the CH Campala *Lr22a* introgression. Jupateco, Yecora 70 and Inia 66 are CIMMYT wheat cultivars. Inia 66 is in the pedigree of CH Campala *Lr22a*

Comparison of SNP density across the chromosome revealed four large regions (haploblocks *a*, *b*, *c* and *d*) with increased SNP density compared to the rest of the chromosome (Fig. 3a). Two of the regions were located on the short arm of the chromosome whereas the largest diverse haploblock of ~ 48 Mb and a shorter fourth haploblock were located towards the telomeric end of the long chromosome arm*.* While the SNP density along most of the chromosome was in the range ~ 27 SNPs/Mb (Fig. 3a), the four diverse haploblocks had SNP densities of 2500–4500 SNPs/Mb. The actual number of polymorphisms might be even higher because SNP calling might not have been possible in many parts of the haploblocks because of the high sequence divergence.

The first haploblock (haploblock *a*) at the distal end of the short chromosome arm contains the *Lr22a* leaf rust resistance gene that was introduced into hexaploid wheat through an artificial hybridization between a tetraploid wheat line and an *Ae. tauschii* accession [44]. There are two genetically distant lineages of *Ae. tauschii*. The D genome of hexaploid wheat was most likely contributed by an *Ae. tauschii* population belonging to lineage 2 [45], whereas the donor of *Lr22a* (*Ae. tauschii* accession RL 5271) belongs to the genetically diverse lineage 1 [46]. The size of the *Lr22a* introgression was subsequently reduced through several rounds of backcrossing with hexaploid wheat and the remaining *Lr22a*-containing segment was bred into elite wheat lines, including CH Campala *Lr22a*, to increase resistance against the fungal leaf rust disease [31]. Based on the SNP density, we were able to estimate the size of the remaining, introgressed *Ae. tauschii* segment to ~ 8 Mb. The original donor of the other three haploblocks (haploblocks *b*, *c* and *d*) could not be traced back and they might be the result of natural gene flow or artificial hybridization. Mapping of independently generated short-read sequences from CH Campala, the recurrent parent that was used to produce the near isogenic line CH Campala *Lr22a*, showed that the same haploblocks were also present in it (Fig. 3a), indicating that these segments were not co-introduced along with the *Lr22a* segment from RL 5271. Haploblock *b* comprised the 285-kb deletion described above (Fig. 1). In particular, the presence of the large continuous haploblock *c* on the long chromosome arm was intriguing. Dot plots allowed us to identify the exact breakpoints of the haploblock (Fig. 3b). While there was high sequence homology in both flanking regions, sequence identity in the intergenic regions broke down inside the haploblock (Fig. 3b). In contrast, dot plots with haploblocks *a*, *b* and *d* revealed a good level of collinearity between Chinese Spring and CH Campala *Lr22a* in intergenic regions despite the increased SNP density (Additional file 1: Figure S1), indicating that haploblock *c* is the most diverse. Comparison to the recently generated high-quality genome assembly of *Ae. tauschii* accession AL8/78 [47], an accession that is closely related to the wheat D genome and that belongs to lineage 2, suggests that haploblock *c* represents an interstitial introgression into CH Campala *Lr22a* (Additional file 1: Figure S2). In Chinese Spring, 723 genes were located in this haploblock, whereas CH Campala *Lr22a* contained 678 genes in this region (Additional file 2: Table S1). The genic sequences in the haploblock region showed a nucleotide sequence identity of 78–100% compared to 99–100% for the genes outside the haploblock. We also observed three inversions of ~ 1.48 Μb, ~ 422 kb and ~ 418 kb in haploblock *c* where the gene order was reversed*.*

To track the possible origin of this introgression, we developed an introgression-specific PCR probe based on the sequence of the left breakpoint in CH Campala *Lr22a*. The marker amplified in several wheat cultivars that were developed by the International Wheat and Maize Improvement Center (CIMMYT) (Fig. 3c). Among them is Inia-66, which is in the pedigree of CH Campala *Lr22a* [48]. These results indicate that the particular segment in CH Campala *Lr22a* might have been introgressed via a CIMMYT cultivar.

### Presence of unique genes and gene synteny

A total of 6018 high confidence (HC) genes were annotated on chromosome 2D of the Chinese Spring reference sequence (IWGSC v1.0) [25] and 5883 HC genes were annotated on chromosome 2D of CH Campala *Lr22a*. A BLASTN analysis of the annotated Chinese Spring genes against the annotated CH Campala *Lr22a* genes produced hits for 5210 out of the 6018 genes, whereas 4656 of the annotated CH Campala *Lr22a* genes produced a BLASTN hit in the annotated Chinese Spring genes. Bi-directional BLAST analysis of the annotated Chinese Spring genes and CH Campala *Lr22a* genes identified a total of 4097 genes that had each other as the top BLAST hit (i.e. groups of paralogs are not included in this dataset).

A total of 808 out of the annotated 6018 HC Chinese Spring 2D genes did not produce any BLAST hit (cut-off E-value 10e-10) against the annotated HC CH Campala *Lr22a* genes, whereas 1227 of the annotated CH Campala *Lr22a* genes did not produce a BLAST hit against the annotated Chinese Spring 2D genes. This would indicate a unique or genotype-specific gene fraction of 13.4 and 20.8% in Chinese Spring and CH Campala *Lr22a*, respectively. However, BLAST analysis of these putatively unique genes against the CH Campala *Lr22a* and Chinese Spring 2D pseudomolecules revealed that 782 of the 808 putatively unique Chinese Spring genes and 1184 of the 1227 putatively unique CH Campala *Lr22a* genes were present on the pseudomolecule. We randomly selected and validated 20 of the 1184 putatively unique CH Campala *Lr22a* genes that produced a BLAST hit on the Chinese Spring 2D pseudomolecule and found intact full-length open reading frames with a 100% sequence identity. Similarly, a random selection of 10 out of the 782 putatively unique Chinese Spring genes revealed that seven genes shared a 100% sequence identity with the respective nucleotide sequence on the CH Campala *Lr22a* 2D pseudomolecule. Hence, these genes were most likely missed or differentially classified (different confidence classes) by the annotation pipeline. In fact, only 26 genes (0.43% of the total genes) were unique to Chinese Spring (genes that did not show BLAST hit against the annotated genes as well as against the pseudomolecule). Of these, 17 fell into the diverse haploblock *c* on the long chromosome arm and two into haploblock *a* on the short arm of the chromosome. In CH Campala *Lr22a*, 43 genes (0.73% of the total genes) were unique, of which 14 were from the diverse haploblock *c* and seven from the *Lr22a* introgression region (haploblock *a*). The unique genes in Chinese Spring and CH Campala *Lr22a* are listed in Additional file 3: Table S2.

There was a high degree of collinearity with only 169 genes that were non-collinear along the 2D chromosome (e.g. the top BLAST hit of the respective gene was not in the syntenic position in the other genotype; Additional file 1: Figure S3). Of the non-collinear genes, two, one, 110 and 11 were from the three diverse haploblocks *a*, *b*, *c* and *d*, respectively. Since the CH Campala *Lr22a* pseudomolecule was produced by anchoring CH Campala *Lr22a* scaffolds to the IWGSC RefSeq v1.0, we only took into account CH Campala *Lr22a* scaffolds that contained two or more genes for the collinearity analysis.

### Chromosome-wide comparison of NLR genes reveals extensive CNV in certain NLR families

**Fig. 4:**
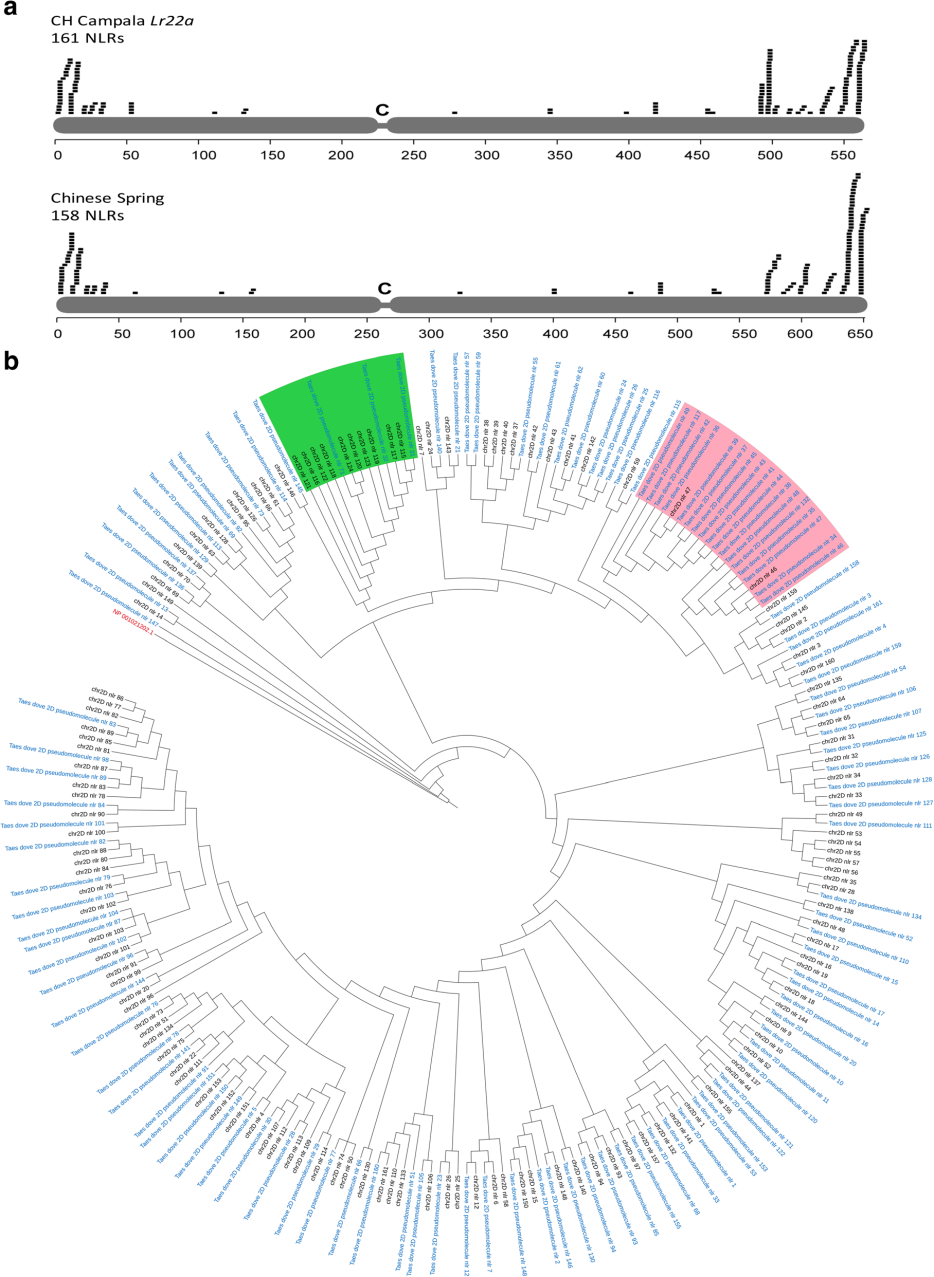
Distribution of predicted NLR genes on chromosome 2D. **a** The x-axis indicates the position in megabases. Note that the scales differ between CH Campala *Lr22a* and Chinese Spring because the sequence assembly of CH Campala *Lr22a* is shorter than that of Chinese Spring. **b** Phylogenetic tree where *blue labels* ‘Taes deove 2D pseudomolecule nlr’ represent the CH Campala *Lr22a* NLRs and *black labels* ‘chr2D nlr’ represent the Chinese Spring NLRs. The two highlighted regions in *green* and *pink* represent chromosomal segments with high copy number variation that are discussed in the text

Regions harbouring NLR genes have been reported to be fast evolving to keep up in the arms race with pathogens [49]. Interestingly, three of the four large InDels identified created CNV for NLR genes. We were therefore interested in the dynamics of chromosomal regions harbouring NLR genes. For chromosome 2D, a total of 161 NLRs were annotated in the wheat line CH Campala *Lr22a* and 158 NLRs for Chinese Spring. The NLRs annotated in the two wheat genotypes showed a high tendency of clustering and they were mostly located in the telomeric regions (Fig. 4a), as is typically found for this gene class [25].

**Fig. 5:**
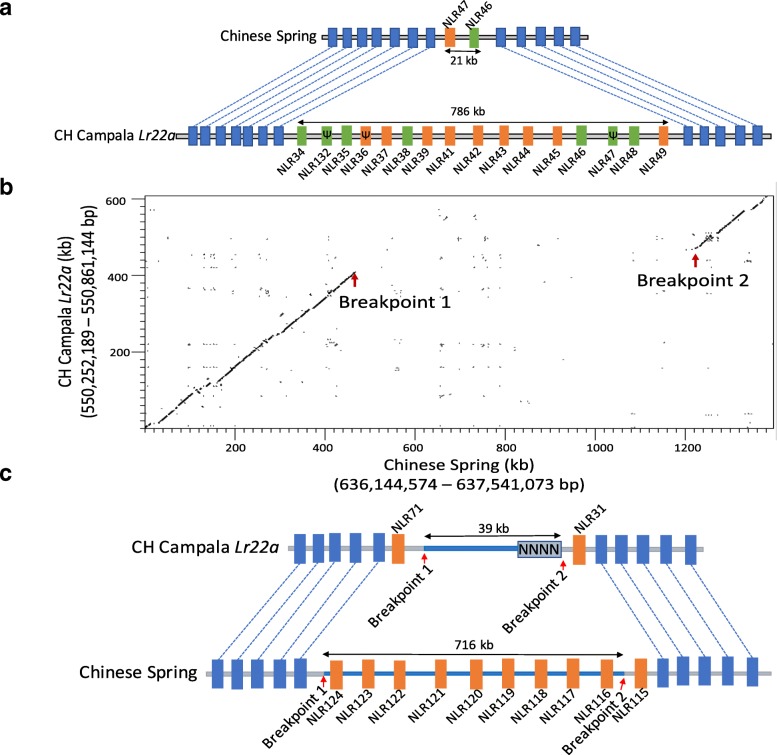
NLR copy number variation. **a** In the CNV1 region we found 16 NLRs in CH Campala *Lr22a* annotated in a 786-kb region. Pseudogenes are marked with *Ѱ*. Chinese Spring has only two NLRs in a 21-kb segment. **b** NLR gene expansion in Chinese Spring. Dot plot of the CNV region between Chinese Spring and CH Campala *Lr22a*. The numbers in brackets refer to the positions of the selected region on the respective pseudomolecule. **c** Chinese Spring had 21 NLRs compared to 14 in CH Campala *Lr22a*, which are shown in *orange* and the collinear genes in the flanking region are shown in *blue*

For CH Campala *Lr22a*, we found that 62 NLR genes resided in seven gene clusters which comprise 38.5% of the total annotated NLRs. The largest cluster contained 19 NLR genes. In Chinese Spring, we found that 71 NLR genes resided in ten clusters which comprise 44.9% of the total annotated NLRs and the largest cluster contained 21 NLRs. A phylogenetic tree revealed that most NLR genes from Chinese Spring had one ortholog in CH Campala *Lr22a* (Fig. 4b). On the other hand, we also observed CNV for certain regions. Two regions, CNV1 and CNV2, were of particular interest because of extensive variation in the NLR copy number between Chinese Spring and CH Campala *Lr22a* (Fig. 4b). In the CNV1 region, CH Campala *Lr22a* had 16 NLR genes annotated in a 786-kb region. The corresponding region in Chinese Spring contained only two NLRs in a 21-kb interval (Fig. 5a). A high degree of gene collinearity flanked the NLR cluster (Fig. 5a). The two NLR copies in Chinese Spring (NLR46 and NLR47) showed 44% sequence identity at the protein level, indicating that they might have arisen from a very ancient gene duplication. The low sequence identity of NLR46 and NLR47 allowed assignment of each of the CH Campala *Lr22a* NLRs to one of the two Chinese Spring copies. This revealed a random pattern, which might be explained by complex duplication and rearrangement events (Fig. 5a). The CNV1 region locates to the diverse haploblock *c*, which might explain the extent of the CNV found in this region.

The CNV2 region affected a segment of ten paralogous NLR genes situated in a 716-kb region in Chinese Spring. In CH Campala *Lr22a*, a 677-kb deletion affected all but two of the NLRs. This CNV2 locates in the collinear region between haploblocks *c* and *d*. For this CNV region we could identify a clear breakpoint at one end whereas the other end had a sequence gap (Fig. 5b, c).

## Discussion

### Molecular mechanisms of structural variations

Different genotypes within a plant species can show tremendous genetic diversity. Beside SNPs, SVs have been identified as a major contributor to phenotypic variation in plants, which is why an understanding of large SVs is of importance for breeding [50]. For example, the durable fungal stem rust resistance gene *Sr2* of wheat was localized to a region on chromosome 3B that showed extensive structural rearrangements between the *Sr2*-carrying wheat cultivar Hope and the susceptible Chinese Spring on an 867-kb chromosome segment [51]. How this structural rearrangement affects the *Sr2*-mediated stem rust resistance is not yet understood. Similarly, large deletions comprising multiple tandemly duplicated transcription factor genes at the *Frost resistance-2* locus are associated with reduced frost tolerance in wheat [52]. While short-read sequencing allowed a comprehensive assessment of genome-wide SNP distributions in cereals [53, 54], the identification of SVs, particularly large InDels, has been challenging due to technical limitations. In wheat, the lack of high-quality chromosome assemblies from multiple genotypes has prevented such comparisons so far. Even for other cereal crop species like rice, maize, barley and sorghum there are no or only very few high-quality de novo assemblies available beside the reference genotypes [22, 55–57]. Here, we compared two high-quality sequence assemblies of bread wheat chromosome 2D that were highly contiguous over megabases, which allowed us to focus on InDels of several hundred kilobases in size. In total, we found that around 0.3% of the chromosome was affected by the four large InDels. Based on these numbers, we estimate that a comparison of any two wheat genotypes would reveal around 30 large InDels affecting ~ 15 Mb across the entire D sub-genome. Not surprisingly, the number of small InDels is much higher than larger structural rearrangements. For example, a comparison of the B73 maize reference assembly to optical maps generated from the two maize inbred lines Ki11 and W22 revealed around 3400 insertions and deletions between two maize lines with an average InDel size of 20 kb [17]. A re-sequencing study in rice revealed a total of 13,045 insertions and 15,151 deletions in the size range of 10–1000 bp [58]. Large InDels affected multiple genes and can therefore have a deleterious effect, particularly in diploid species.

Unequal crossing over and DSB repair were identified as the molecular mechanisms responsible for large InDels in our study. Analyses in *Brachypodium* revealed that DSB repair is the most common mechanism for structural rearrangements [34, 59]. The error prone DSB repair leads to insertions, deletions or rearrangements in the genome. In our comparative analysis, we found a large deletion of 494 kb in Chinese Spring where DSB repair via single strand annealing led to the deletion of the intervening region between the conserved motifs known as DSB signatures. Similar mechanisms were identified in a comparative analysis of the two barley cultivars Barke and Morex, where DSB repair accounted for 41% of the InDel events [33]. DSB repair signatures were also found in maize where they flanked small InDels ranging from 5 to 175 bp [60]. Apart from DSB repair, another frequently observed mechanism for SV is unequal crossing over. We found a 285-kb deletion in Chinese Spring where the deletion was a result of an improper alignment of two highly similar NLR genes that served as a template for unequal crossing over. Unequal crossing over has been shown to be one of the main driving forces for genome differences and has been reported to occur in various disease resistance gene families where they result in novel specificities and haplotypes [37]. For example, unequal crossing over between homologs in the maize rust resistance locus *Rp1* led to the formation of recombinant genes with diverse resistance specificities [61, 62]. In soybean, unequal crossing over at the *RPS* locus was associated with loss of resistance to *Phytophthora* due to the deletion of a NLR*-*like (*NBSRps4/6*) sequence [63].

### Identification of diverse haploblocks—implications for wheat D genome dynamics

In addition to SVs, the chromosome-scale assemblies also allowed us to assess SNP density across the entire chromosome and to identify large contiguous blocks with strong variation from the average SNP density. This revealed the presence of four haploblocks that showed a much higher SNP density compared to the rest of the chromosome. One of these haploblocks (haploblock *a*) could be traced back to an artificial introgression that carries the adult plant leaf rust resistance gene *Lr22a* [32, 64]. *Lr22a* was introgressed into hexaploid wheat by artificially hybridizing the tetraploid wheat line tetra-Canthatch with the diploid *Ae. tauschii* accession RL 5271 [44]*.* The crossing of tetraploid wheat with diverse *Ae. tauschii* accessions results in so-called synthetic wheat. This is a widely explored strategy in breeding to compensate for the loss of diversity in hexaploid wheat that went along with domestication and modern breeding [65–67]. After this initial cross, the resulting synthetic hexaploid wheat line was backcrossed six times with the historically important North American wheat cultivar Thatcher, which resulted in the *Lr22a*-containing backcross line ‘Thatcher *Lr22a*’ (RL 6044). This backcross line then served as the donor to transfer *Lr22a* into elite wheat cultivars, including the Canadian wheat cultivar ‘AC Minto’ and the Swiss spring wheat line CH Campala *Lr22a* [31, 64]. The SNP density analysis allowed us now to infer the size of the remaining RL 5271 segment after a limited number of crosses. We did not find evidence for co-introduction of additional segments from the original *Ae. tauschii* donor along chromosome 2D. More interestingly, three additional diverse haploblocks (haploblocks *b*, *c* and *d*) of almost 9, 48 and 4 Mb were identified towards the telomeric end of the short and long chromosome arms, respectively. It has been reported that the wheat D genome was most likely contributed by an *Ae. tauschii* population from a region close to the southern or southwestern Caspian Sea. This accession belonged to one of two genetically distinct sublineages within the *Ae. tauschii* gene pool (sublineage 2) [45]. However, it has been found that gene flow from *Ae. tauschii* accessions belonging to the genetically distant sublineage 1 occurred after the formation of hexaploid wheat, which might explain the presence of contiguous haploblocks with increased diversity. Interestingly, Wang et al. [45] identified a putative introgression of *Ae. tauschii* sublineage 1 on the telomeric end of chromosome arm 2DL in hexaploid wheat, which might be identical to the diverse haploblock *c* identified in our study. Alternatively, these diverse haploblocks might stem from an alien introgression from another grass species. Interspecies hybridizations are a common method in wheat breeding to transfer specific traits from wild and domesticated grasses into wheat [68]. In contrast to the naturally occurring gene flow from *Ae. tauschii*, the vast majority of these alien introgressions were artificially produced and require in vivo culture techniques like embryo rescue. The length of the haploblock *c* was surprising because the size of haploblocks is expected to be negatively correlated with recombination rates [69]. Since the haploblock *c* located to the highly recombining telomeric end of the chromosome, we would expect that its size decreases over time. One explanation for conservation of this haploblock could be that its presence suppresses recombination in this area. In contrast to haploblocks *a*, *b* and *d*, we observed a breakdown of sequence homology in intergenic regions in haploblock *c*. On the other hand, the gene order was largely collinear in haploblock *c*, which should be sufficient for recombination in this chromosome segment. A second explanation is that this haploblock *c* might be widely present in the wheat gene pool or in particular breeding programs. For example, PCR analysis revealed that the haploblock *c* was present in multiple CIMMYT wheat lines. This would allow recombination in the haploblock without decreasing its size. In summary, a considerable fraction of the chromosome (10%) was made up of haploblocks with a much greater diversity than the rest of the chromosome. This highlights the importance of natural gene flow and artificial hybridization as sources for diversity in cereal breeding.

### Comparative genomics: Real differences vs artefacts—a note of caution

In addition to a better understanding of genome dynamics, our analysis also revealed that manual inspection of variation revealed by automated scripts is required in order to distinguish true variants from assembly or annotation artefacts. For example, 22 of the 26 initially identified large InDels had Ns at both ends, indicating that they were most likely due to mis-assembly in one or the other genotype. A similar observation was made for the gene annotation. Our initial comparison of annotated genes revealed a high proportion (14–21%) of genes that were uniquely present in only one of the two wheat genotypes. Careful validation of the data, however, revealed that most of these genotype-specific genes produced a BLAST hit at the syntenic position in the other wheat line, indicating that these genes are present but that they were most likely missed or differentially classified (high and low confidence classes) by the annotation pipeline. Potential reasons for this observation include artefacts and errors while aligning gene evidence and predicting gene structures, conflicting transcriptome evidence and truncated or incomplete gene models. The actual fraction of unique genes was considerably lower with only 26 and 43 genes that were truly unique in Chinese Spring and CH Campala *Lr22a*, respectively. A recent pan-genome study that was based on short-read resequencing of 18 wheat cultivars compared to a medium-quality Chinese Spring assembly reported a total of 128,656 genes in the genome of hexaploid wheat, of which 49,952 (38.8%) were variable [30]. On chromosome 2D, 3.3–11% of the 4703 annotated genes in the respective Chinese Spring assembly were reported to be absent in the other wheat cultivars. Similarly, Liu et al. [29] mapped Illumina reads of flow-sorted chromosome 3B of the *Fusarium* crown rot-resistant wheat line CRNIL1A to a high-quality assembly of chromosome 3B from Chinese Spring [12]. They identified 499 gene-containing contigs that were specifically found in CRNIL1A but absent in Chinese Spring. The respective Chinese Spring assembly that was used for the comparison contained 5326 protein-coding genes and, hence, the unique gene fraction in CRNIL1A was estimated to be 9.4%. Surprisingly, our conservative approach revealed that the fraction of unique genes is in the range of 0.43–0.73% only, which is 5–25-fold lower than the estimates that were based on short-read resequencing. It is possible that Chinese Spring and CH Campala *Lr22a* share a particularly high degree of sequence identity on chromosome 2D compared to other cultivars, although there is no obvious connection between the two wheat lines based on the pedigree information. It is, therefore, more likely that the number of unique genes was overestimated in previous studies, which might have been caused by assembly or annotation artefacts that could not have been accounted for. It has been proposed that the quality of an assembly does affect the quality of gene annotation [70]. An example for this is the maize line B73, for which two high-quality de novo genome assemblies exist. While the first version of the reference sequence predicted 32,540 protein coding genes in the B73 genome [57], a recently released and improved version of the same genotype reported 39,324 protein coding genes [17]. The difference of 6784 genes (17%) can only be explained by technical variation. This example highlights the fact that the assembly quality and annotation procedure can have a tremendous influence on the prediction of the gene content and, hence, the estimation of genotype-specific genes. In summary, we provide evidence that the number of unique or variable genes in wheat has been overestimated in past studies due to low assembly quality and intrinsic variation in genome annotation pipelines. Hence, the so called pan-genome of wheat might be considerably smaller than what was previously estimated [30]. It has to be noted that the wheat D genome is the least diverse of the three wheat sub-genomes [3, 6] and it is likely that the fraction of unique genes is higher in the A and B genomes, although most likely not as high as estimated previously. A recent study in *Arabidopsis thaliana* also found that careful manual curation is necessary in order to avoid overestimation of genotype-specific genes. The comparison of high-quality assemblies of the *Arabidopsis* ecotypes Columbia and Landsberg revealed 63 (0.23%) unique genes in Columbia and 40 (0.14%) unique genes in Landsberg, which is very similar to the numbers we report in our comparison [71]. A comparison of two high-quality assemblies of the *indica* rice lines Zhenshan 97 and Minghui 63 revealed around 4% genotype-specific genes. An important note is that these calculations focused on the presence–absence variation of single genes and did not measure the extent of gene copy number variation as it was, for example, described for the NLR genes in our study.

## Conclusions

This study provides the first comparison of two wheat pseudomolecules based on high-quality de novo chromosome assemblies. The megabase-sized scaffolds allowed us to focus particularly on InDels several hundred kilobases in size. Our analysis revealed that around 0.3% of the chromosome was affected by large InDels between the two wheat lines. Our study also revealed that careful manual validation is required in order not to overestimate the frequency of InDels and genotype-specific genes. In particular, 84% of the InDels that were initially identified and 96% of the genotype-specific genes identified through automated pipelines were removed after manual curation because they were most likely due to assembly and annotation artefacts. It is conceivable that previous comparative analyses in wheat that were based on short-read resequencing alone could not account for these problems. We therefore highlight the importance of manual data validation in future wheat pan-genome projects.

## Methods

### CH Campala *Lr22a* pseudomolecule assembly

The initial sequence assembly provided by Dovetail Genomics consisted of 10,344 sequence scaffolds (hereafter referred to as Dovetail scaffolds) with an average size of 54.8 kb and an N50 of 9.758 Mb [32]. To anchor these scaffolds to the IWGSC RefSeq v1.0 chromosome 2D, segments of the scaffolds were used in BLASTN searches against the Chinese Spring chromosome [25]. Dovetail scaffolds shorter than 10 kb were used in their entirety for the BLASTN search. For Dovetail scaffolds between 10 and 200 kb, a 1-kb segment every 30 kb was used for the BLASTN search. For Dovetail scaffolds larger than 200 kb, a 1-kb segment every 100 kb was used for BLASTN search. For each Dovetail scaffold, it was then determined where the majority of BLAST hits were located in Chinese Spring 2D. Based on this information, Dovetail scaffolds were ordered.

After sequence scaffolds were assembled into a first version of a pseudomolecule, we searched for large-scale breaks in gene collinearity when compared to Chinese Spring chromosome 2D. Here, we focused on blocks of BLASTN hits that mapped to completely different regions of the genome. If the end of a non-collinear block coincided with the end of a Dovetail scaffold, this was interpreted as an assembly artefact. The approximate location of the mis-assembly was identified and the respective Dovetail scaffold was then split into segments. We identified ten putatively chimeric Dovetail scaffolds with assembly errors. These were split into 24 segments (some Dovetail scaffolds contained multiple mis-assemblies) which were then anchored individually to Chinese Spring chromosome 2D.

A total of 7617 Dovetail scaffolds were integrated to the final pseudomolecule of 563 Mb, representing 73% of all Dovetail scaffolds and 98.92% of the total length of the Dovetail assembly. The integrated 7617 Dovetail scaffolds have an N50 of 8.78 Mb and an N90 of 1.89 Mb. The scaffold N50 of 8.78 Mb is slightly lower than the N50 of the original assembly obtained from Dovetail Genomics, which is due to the splitting of chimaeric scaffolds.

### Gene annotation

We combined two strategies to facilitate gene prediction on the CH Campala *Lr22a* 2D pseudomolecule: prediction using homology from reference proteins and prediction using gene expression data.

For the homology-based annotation step, we combined available Triticeae protein sequences obtained from UniProt (05/10/2016), which contain among others validated protein sequences from *Triticum aestivum*, *Aegilops tauschii* and *Hordeum vulgare.* These protein sequences were mapped to the nucleotide sequence of the CH Campala *Lr22a* 2D pseudomolecule using the splice-aware alignment software Genomethreader (version 1.6.6; arguments -startcodon -finalstopcodon -species rice -gcmincoverage 70 -prseedlength 7 -prhdist 4) [72].

In the expression data-based step, we used full-length cDNA sequences (leaf, root, seedling, seed, spike and stem [14] and one full-length cDNA library), as well as multiple RNASeq datasets (E-MTAB-2127, SRP045409, ERP004714/URGI, E-MTAB-21729, PRJEB15048) as evidence to guide the gene structure prediction on the CH Campala *Lr22a* 2D pseudomolecule. Full-length cDNA and IsoSEQ nucleotide sequences were aligned to the pseudomolecule using GMAP (version 2016-06-30, standard parameter, PMID 15728110), whereas RNASeq datasets were first mapped using Hisat2 (version 2.0.4, parameter --dta, PMID 25751142) and subsequently assembled into transcript sequences by Stringtie (version 1.2.3, parameters m 150 -t -f 0.3, PMID 25690850). All transcripts from flcDNA, IsoSeq and RNASeq were combined using Cuffcompare (version 2.2.1, PMID 26519415) and merged with Stringtie (version 1.2.3, parameters --merge -m 150) to remove fragments and redundant structures. Next, we used Transdecoder (version 3.0.0) to find potential open reading frames and to predict protein sequences. We used BLASTP (ncbi-blast-2.3.0+, parameters -max_target_seqs 1 -evalue 1e-05, PMID 2231712) to compare potential protein sequences with a trusted protein reference database (Uniprot Magnoliophyta, reviewed/Swissprot, downloaded on 3 Aug 2016) and used hmmscan (version 3.1b2, PMID 22039361) to identify conserved protein family domains for all potential proteins. BLAST and hmmscan results were fed back into Transdecoder-predict to select the best translations per transcript sequence.

Finally, all results were combined and redundant protein sequences were removed to form a single non-redundant candidate dataset. In order to differentiate candidates into complete and valid genes, non-coding transcripts, pseudogenes and transposable elements, we applied a confidence classification protocol. Candidate protein sequences were compared against the following three manually curated databases using BLAST: first, PTREP, a database of hypothetical proteins that contains deduced amino acid sequences in which, in many cases, frameshifts have been removed, which is useful for the identification of divergent TEs having no significant similarity at the DNA level; second, UniPoa, a database comprised of annotated Poaceae proteins; third, UniMag, a database of validated magnoliophyta proteins. UniPoa and UniMag protein sequences were downloaded from Uniprot on 30 Aug 2016 and further filtered for complete sequences with start and stop codons. Best hits were selected for each predicted protein to each of the three databases. Only hits with an E-value below 10e-10 were considered.

Furthermore, only hits with subject coverage (for protein references) or query coverage (transposon database) above 90% were considered significant and protein sequences were further classified using the following confidence: a high confidence (HC) protein sequence is complete and has a subject and query coverage above the threshold in the UniMag database (HC1) or no blast hit in UniMag but in UniPoa and not TREP (HC2); a low confidence (LC) protein sequence is not complete and has a hit in the UniMag or UniPoa database but not in TREP (LC1), or no hit in UniMag and UniPoa and TREP but the protein sequence is complete.

The tag REP was assigned for protein sequences not in UniMag and complete but with hits in TREP.

In a last step, a set of representative genes within the HC group was selected by choosing the longest transcript for each predicted gene model.

### NLR annotation and phylogenetic tree

NLR loci on the CH Campala *Lr22a* pseudomolecule were annotated using NLR-Annotator [73]. The initial fragmentation step of NLR-Annotator was performed generating 20-kb fragments that overlap by 5 kb. Multiple alignments of NB-ARC associated amino acid motifs were generated using NLR-Annotator (output option –a). Multiple alignment files were concatenated and a comparative phylogenetic tree was generated using FastTree [74] version 2.1.7 [75].

### Identification of the SVs

We analyzed SVs in the telomeric and interstitial regions and excluded the centromeric region, which was ~ 100 Mb in size (position 190–290 Mb in Chinese Spring pseudomolecule and 150–250 Mb in CH Campala *Lr22a* pseudomolecule). The centromeric region is extremely repetitive and gene-poor and alignments were difficult. For the identification of the SVs, we segmented the Chinese Spring and CH Campala *Lr22a* pseudomolecules in windows of 10 Mb and performed dot plot alignments (program DOTTER) [76]. For each of the InDels observed, we analyzed the sequence alignments to identify the region where the sequence similarity broke down and this region was called the breakpoint. We spliced out 5-kb sequence upstream and downstream of these breakpoints and performed BLASTN searches [77] against the repeat database to identify transposable elements and also against the *Brachypodium distachyon* coding sequence database [78] to identify genes in the flanking regions to understand the molecular mechanism underlying the observed SVs.

To identify NLR CNV, we compared the NLR clusters in Chinese Spring and CH Campala *Lr22a* and identified the breakpoints as described above*.* The sequences upstream and downstream of breakpoints were used to identify the collinear genes using BLAST search against the annotated CH Campala *Lr22a* and Chinese Spring genes. Putative start and stop codons of the annotated NLRs were identified based on the orthologs of these NLRs in *Brachypodium distachyon.* The coding sequences of these *Brachypodium distachyon* NLRs were taken from the *Brachypodium distachyon* coding sequence database [78] and were used for the dot plot alignment to identify the coding sequence of the Chinese Spring and CH Campala *Lr22a* NLRs. Pseudogenes were predicted on the basis of frameshift mutations, premature stop codon or insertion of a transposable element resulting in a pseudogene.

### Haploblock analysis and validation

For the identification of the haploblock region, we mapped previously generated Illumina reads of CH Campala *Lr22a* and CH Campala [32] to the Chinese Spring pseudomolecule using CLC Main Workbench 7 (Qiagen) with standard parameters. The mapped read file was later used for the variant call analysis by CLC Main Workbench 7 (Qiagen) using standard parameters. SNP density was calculated in sliding windows of 2.5 Mb. To verify the haploblock *c* region we designed a PCR probe (forward primer GCCACGAGCGTGGTCGTG, reverse primer CCTTCATAGCTCCGTAGAAG) spanning the left border of the haploblock *c* of CH Campala *Lr22a*. The PCR amplification was performed in 20 μl reaction mixture containing 65 ng of genomic DNA, 1 μl of 2.5 mM dNTP’s, 1 μl of 10 μM of each primer and 0.25 units of Sigma Taq polymerase at 60 °C annealing temperature for 35 cycles. The cycling parameters used were pre-denaturation at 95 °C for 4 min, which was followed by 35 cycles of 95 °C for 30 s, annealing at 60 °C for 30 s, 72 °C for 2 min and a final extension at 72 °C for 10 min. The PCR products were separated on 1.0% agarose gels.

## Additional files

Additional file 1: **Figure S1.** Haploblocks *a*, *b* and *d* show sequence homology in the intergenic regions between Chinese Spring and CH Campala *Lr22a* (PDF). **Figure S2.** Dot plot of the haploblock *c* region from Chinese Spring and CH Campala *Lr22a* with *Ae. tauschii*. **Figure S3.** Gene collinearity between Chinese Spring and CH Campala *Lr22a*. (PDF 3408 kb)

Additional file 2: **Table S1.** List of 678 CH Campala *Lr22a* genes that were found in haploblock *c*. (XLSX 32 kb)

Additional file 3: **Table S2.** Unique genes identified in Chinese Spring and CH Campala *Lr22a*. (XLSX 14 kb)
