# Supplementary material for: Chromosome-scale comparative sequence analysis unravels molecular mechanisms of genome dynamics between two wheat cultivars
Source: Genome Biol. 2018 Aug 17;19:104. doi: 10.1186/s13059-018-1477-2 (PMC6097286; doi:10.1186/s13059-018-1477-2)

Additional file 1: Fig. S1. Haploblocks *a, b* and *d* showed sequence homology in the intergenic regions between Chinese Spring and 'CH Campala *Lr22a*'. **a** Dot plot of haploblock *a* with the flanking region represents the *Lr22a* introgression of ~8 Mb in size. **b** Dot plot of ~9 Mb haploblock *b* with the flanking region. **c** Dot plot of the ~4 Mb haploblock *d* with the flanking region. The numbers in brackets refer to the positions of the selected region on the respective pseudomolecule.

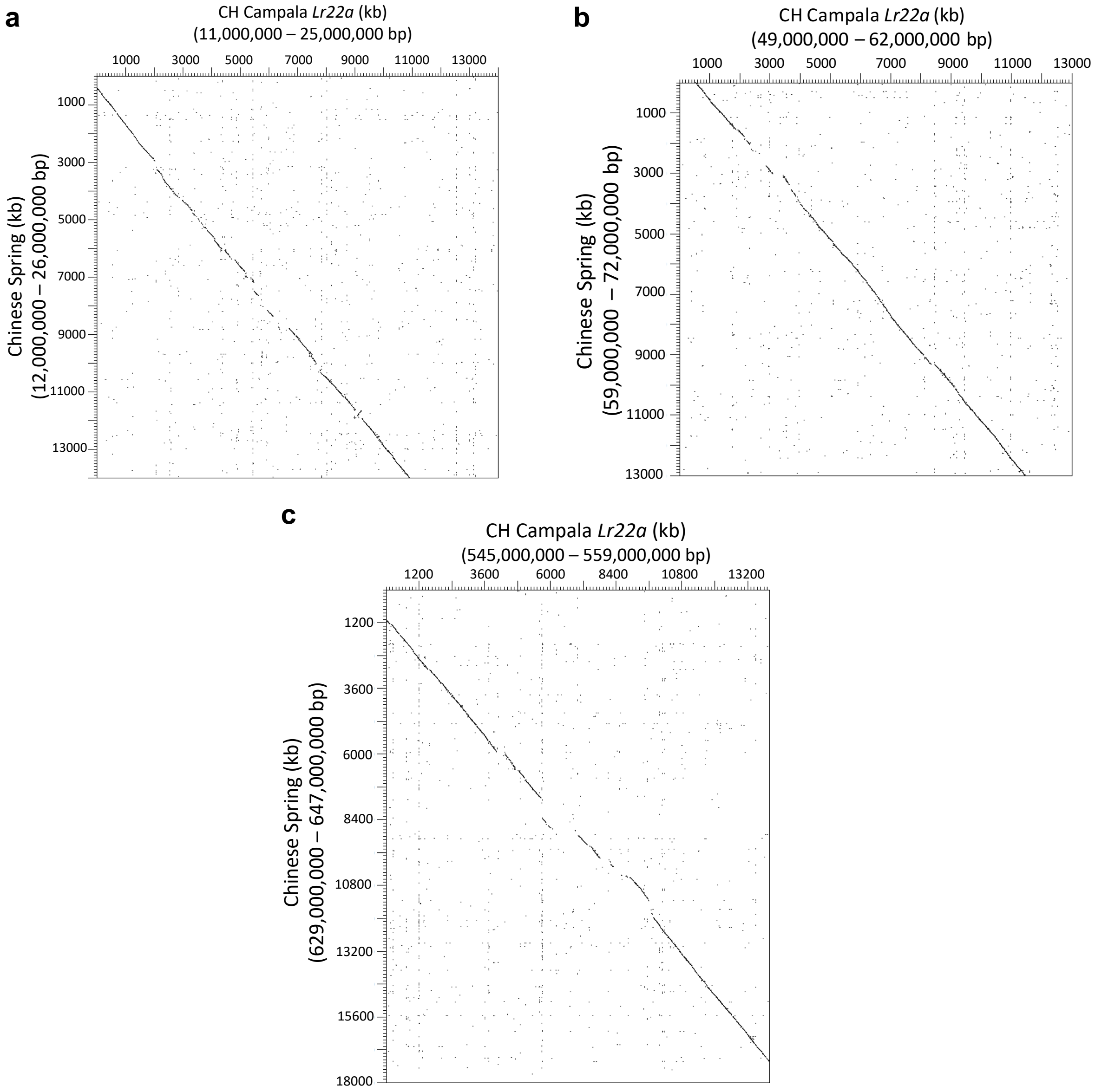

Additional file 1: Fig. S2. Dot plot of the haploblock *c* region from Chinese Spring and 'CH Campala *Lr22a*' with *Ae. tauschii*. **a** Dot plot of 5 Mb region upstream and 10 Mb downstream of breakpoint 1 of 'CH Campala *Lr22a*' and Chinese Spring with *Ae. tauschii.* **b** Dot plot of 5 Mb region upstream and 10 Mb downstream of breakpoint 2 of 'CH Campala *Lr22a*' and Chinese Spring with *Ae. tauschii.* The numbers in brackets refer to the positions of the selected region on the respective pseudomolecule.

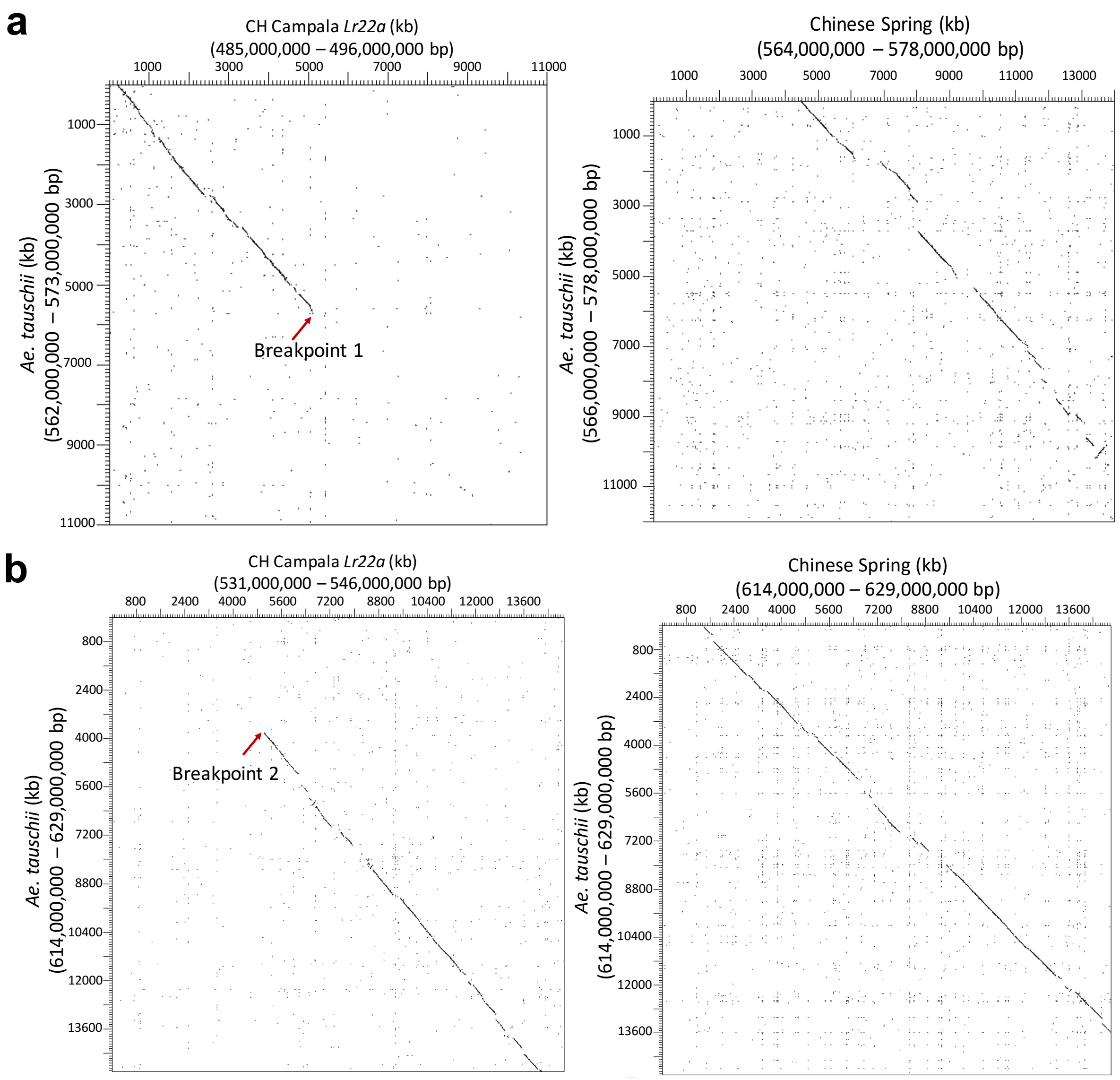

Additional file 1: Fig. S3. Gene collinearity between Chinese Spring and 'CH Campala *Lr22a*'. Collinear genes are connected with black lines and non-collinear genes are connected with red lines. For better visibility, only every fifth gene is displayed. The purpose is to illustrate that the vast majority of genes are in perfectly collinear order. At the centromeric region (190-290 Mb in Chinese Spring and 150-250 Mb in 'CH Campala *Lr22a*'), the gene density is low but they show good collinearity.

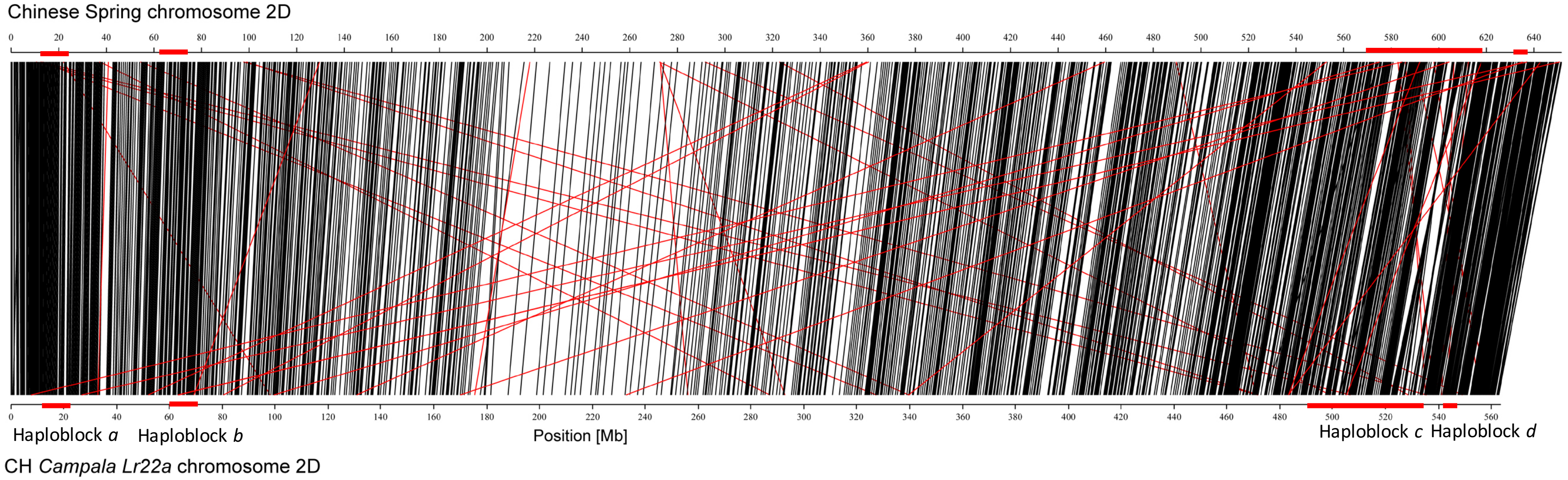

Supplement: Supplementary file 1 — Figure S1. Haploblocks a, b and d show sequence homology in the intergenic regions between Chinese Spring and CH Campala Lr22a (PDF). Figure S2. Dot plot of the haploblock c region from Chinese Spring and CH Campala Lr22a with Ae. tauschii. Figure S3. Gene collinearity between Chinese Spring and CH Campala Lr22a. (PDF 3408 kb) [file 13059_2018_1477_MOESM1_ESM.pdf]
